# Explainable Machine Learning for Predicting Student Depression Risk

**DOI:** 10.3390/bioengineering13091083

**Published:** 2026-09-18

**Authors:** Reynalyn Cernechez, Seyed Ebrahim Hosseini, Muhammad Nadeem, Shahbaz Pervez, Mohammad Salah, Muazma Shahbaz

**Affiliations:** 1 School of Applied IT, Whitecliffe, Auckland 1010, New Zealand; 20250811@mywhitecliffe.com (R.C.); shahbazp@whitecliffe.ac.nz (S.P.); muazmas@whitecliffe.ac.nz (M.S.); 2 College of Engineering and Technology, American University of the Middle East, Egaila 54200, Kuwait; muhammad.nadeem@aum.edu.kw (M.N.); mohammad.salah@aum.edu.kw (M.S.)

**Keywords:** student depression risk, non-clinical data, machine learning, explainable artificial intelligence, fairness evaluation, decision support system

## Abstract

Depression among students has emerged as a critical issue within educational institutions, leading to the need for approaches that can support early identification of students at risk. This study developed an explainable machine learning framework for predicting student depression risk using non-clinical demographic, academic, lifestyle, and psychosocial factors. Using a public OpenML dataset containing approximately 27,901 student records, logistic regression, random forest, and XGBoost models were developed and evaluated using accuracy, precision, recall, F1-score, and ROC-AUC. Fairness evaluation was conducted across gender and financial stress groups, while SHAP and LIME were applied to provide global, class-level, and local explanations of model predictions. Results showed that all models achieved strong predictive performance, with XGBoost performing best (accuracy = 0.846, recall = 0.883, F1-score = 0.870, ROC-AUC = 0.920). The fairness evaluation showed relatively consistent performance across gender groups, while models performed better in identifying depression cases among students in the high financial stress group. Further, explainability analysis identified Suicidal Thoughts, Academic Pressure, and Financial Stress as the most influential predictors. A Streamlit prototype was developed to demonstrate practical deployment. Overall, the findings demonstrate the potential of explainable machine learning to support student depression risk identification and provide interpretable model predictions.

## 1. Introduction

Student depression has been one of the major concerns in the context of education due to its influence on students’ academic performance, motivation, participation, and overall wellbeing [1,2]. Recent research demonstrated high prevalence of depression among students and, thus, highlighted the need for approaches that can help in early detection of students with depression [3,4,5]. School-based mental health screening can support early identification of students who may require additional support, although its implementation may involve practical challenges such as staff training, consent procedures, and addressing concerns surrounding mental health screening [6,7,8]. Research has shown that student depression is influenced by a combination of different factors such as academic, demographic, lifestyle, and psychosocial factors, including academic pressure, financial stress, sleep habits, and family-related influences [9,10,11]. This complex nature requires approaches that consider these factors together rather than in isolation.

This need has encouraged the growing application of machine learning techniques capable of analyzing large and multidimensional student datasets, including approaches for detecting and predicting stress and stress-related mental disorders [12]. Recent studies have demonstrated that non-clinical student information can be used to develop predictive models with strong performance, supporting the potential of machine learning as a tool for early risk identification in educational settings [9,11,13,14]. This method is also feasible since clinical methods like PHQ-9, DASS-21, or GAD-7 can trigger issues regarding label leakage, generalizability, and deployment within schools. There have also been successful applications using different algorithms, such as logistic regression, random forest, XGBoost, ensemble methods, and deep learning methods, in predicting student depression [15,16,17,18]. These studies have revealed that it is possible to achieve relevant predictive performance without depending solely on clinical measures, thereby emphasizing the potential role of machine learning as a tool for promoting wellbeing initiatives among students.

As the development of machine learning in this area has evolved, recent research has increasingly focused on more than just prediction. For instance, studies have used XAI methods such as SHapley Additive exPlanations (SHAPs) and Local Interpretable Model-agnostic Explanations (LIMEs) to enhance the interpretability of model predictions [9,19]. At the same time, fairness has also been considered important for the models since high performance of a model does not always mean that it performs equally well on different demographic or socioeconomic groups [20,21,22]. Previous studies have also evaluated model performance across demographic and social subgroups, with some reporting relatively consistent performance across these groups [23]. These developments can be seen as part of the growing trend toward responsible machine learning, in which transparency, fairness, accountability, and usability have become additional features besides prediction performance [24,25].

However, even with all these developments, there is no full incorporation of all these elements into the pipeline. In many cases, research is focused on predictive performance rather than on other concerns, such as explainability, fairness, or deployment. Some studies incorporate interpretability techniques, while some check fairness but not explainability or deployment. Despite the many valuable contributions made by earlier researchers towards understanding the problem of predicting student depression, relatively few studies have managed to combine elements of predictive modeling, fairness evaluation, explainability analysis, and practical deployment within a single framework. Thus, there remains the need to design an approach that will not only enable prediction but will also provide interpretability, responsible evaluation, and practical application [2,22,24,25].

This study is grounded in the theoretical framework of Social Determinants of Mental Health, which acknowledges that mental health is determined by various demographic, academic, lifestyle, and psychosocial factors. Additionally, the Statistical Learning Theory, Fairness in Machine Learning, Explainable AI, and Ngā Tikanga Paihere were instrumental in developing the framework in terms of modeling generalization, subgroup evaluation, and ensuring responsible data usage. These theories have been considered while designing the proposed framework for predicting depression risk among students.

In order to fill the gap, this study designed and analyzed an explainable machine learning framework for predicting the risk of student depression based on demographic, academic, lifestyle, and psychosocial non-clinical factors. Logistic regression, random forest, and XGBoost have been developed and analyzed using an OpenML public dataset with approximately 27,901 students’ data. Post hoc fairness evaluation was performed on logistic regression and XGBoost in terms of gender and financial stress subgroups, whereas SHAP and LIME were employed to provide global, class-level, and local explanations of the model’s outputs. Moreover, a prototype based on Streamlit was developed to demonstrate the feasibility of integrating the model and its explanations into a web-based decision-support tool.

The findings showed that all three models had high levels of prediction capabilities, with XGBoost producing the strongest overall results. Fairness evaluation showed rather consistent performance for gender groups but higher variability for financial stress groups. Explainability analysis revealed Suicidal Thoughts, Academic Pressure, and Financial Stress to be the strongest predictors, with Age, Work/Study Hours, Degree, and Study Satisfaction also made moderate by meaningful contribution. Overall, the results demonstrate how predictive modelling, fairness evaluation, explainability, and prototype development can be integrated into a single framework to support more interpretable approaches to student depression risk prediction.

## 2. Materials and Methods

### 2.1. Study Design and Workflow

**Figure 1:**
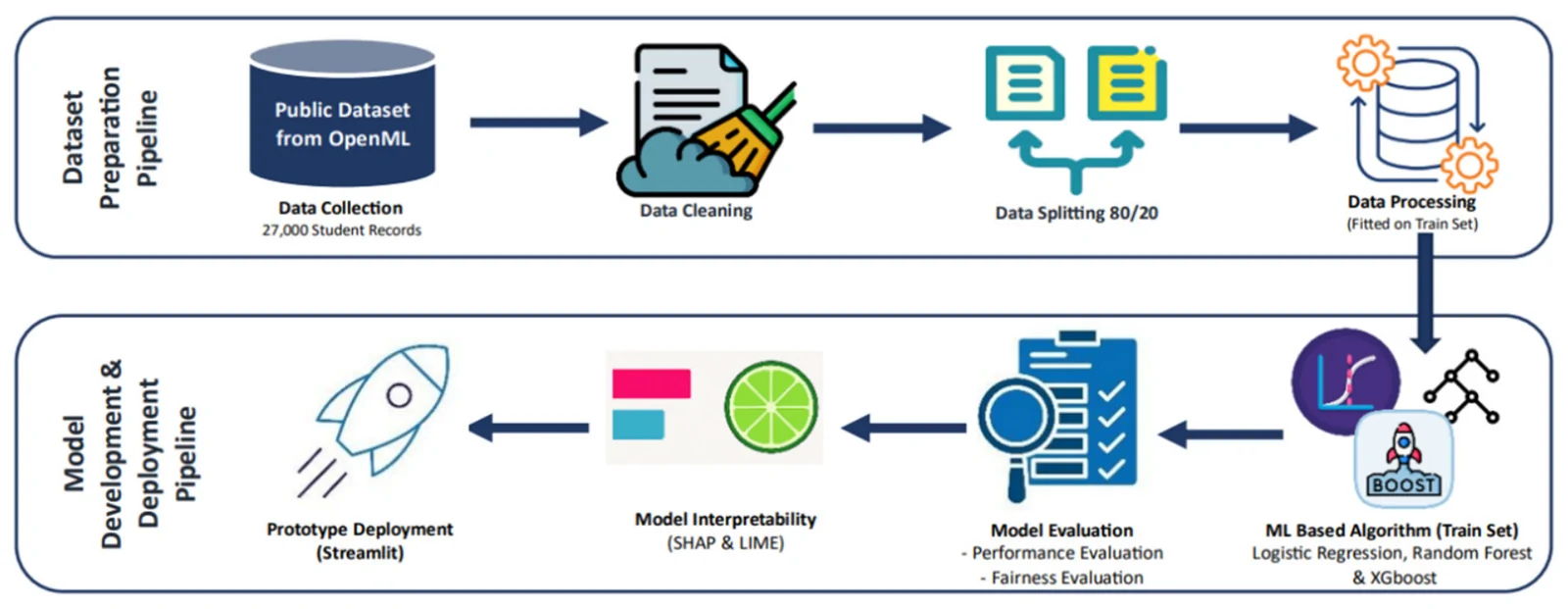
Workflow of the proposed explainable machine learning framework for student depression risk prediction. Arrows indicate the direction of the workflow from data preparation to prototype deployment.

A quantitative machine learning approach has been used in the study to develop an explainable framework for student depression prediction based on non-clinical student data. The framework process includes data preparation, feature selection, model development, performance evaluation, fairness assessment, explainability analysis, and finally, developing a prototype. Three machine learning models—logistic regression, random forest, and XGBoost—have been developed and evaluated to identify the most suitable model. For fairness evaluation, gender and financial stress groups have been analyzed, and SHAP and LIME have been used to explain the model at global and local explanations. Lastly, the best-performing model was integrated into a Streamlit (version 1.45.1) prototype to show practical deployment and interpretability. Figure 1 shows the overall workflow of the proposed framework.

To further formalize the methodological workflow, Algorithm 1 demonstrates the step-by-step procedure used in this study, starting from data inspection and preprocessing to model training, evaluation, fairness evaluation, explainability analysis, and prototype deployment.
**Algorithm 1:** Methodological workflow for non-clinical student depression risk prediction, fairness evaluation, explainability analysis, and prototype deployment.Input:Feature matrix X and target variable yOutput:Best-performing model M* and prediction/explanatiom outputsStep 1:Initial Data InspectionStep 2:Data Cleaning and FilteringStep 3:Removal of Non-Predictive and Sparse FeaturesStep 4:Exploratory Data Analysis (EDA)Step 5:Split X and y into training and test sets using a stratified 80:20 splitStep 6:Fit preprocessing pipeline steps on X_train-impute missing values (numerical = median) and (categorical = most frequent/mode)-standardise numerical features-OHE categorical featuresStep 7:Train and tune Logistic Regression, Random Forest, and XGBoost using 5-fold crossvalidation on the training set with preprocessing fitted in pipelineStep 8:Evaluate all models on the held-out test set using Accuracy, Precision, Recall, F1-score, and ROC-AUCStep 9:Conduct post-hoc fairness evaluation of M* and Baseline across gender groups and financial stressStep 10:Apply SHAP and LIME to interpret model behaviour and individual predictions for M*Step 11:Integrate M* and explanation outputs into the Streamlit prototype with SHAP explanation

### 2.2. Dataset

A publicly accessible OpenML dataset published by Jurado, I. C. was used in this study. The dataset contains approximately 27,901 anonymized student records collected from students across 52 cities in India and included demographic, academic, lifestyle, and psychosocial features with depression as the binary target variable. The target variable represents a binary depression outcome (Yes/No). A subsequent peer-reviewed study using the same dataset describes the data as originating from anonymized, self-reported surveys distributed across educational institutions [9]. However, the available OpenML documentation does not provide detailed information about the original sampling frame, specific data-collection sites, collection period, survey instrument, consent procedures, or the precise procedure used to derive the binary depression outcome. Therefore, the binary target is treated in this study as a dataset-defined outcome used for risk identification and decision support rather than as a clinical diagnosis. This finding is in agreement with the results of earlier studies which found that academic, lifestyle, and psychosocial variables are valuable predictors of depression [11,14,19]. The dataset showed a slight class imbalance, with 58.5% of records labelled as positive for the depression outcome and 41.5% labelled as negative.

The dataset was treated as secondary data. It did not involve any direct interaction with any participant, intervention, or personal data collection conducted. No participants were recruited or directly involved in the present study, and no new personal data were collected. The variables included in the study are summarized in Table 1. With the dataset collected from a specific student population, there is a possibility that the results are not applicable to other institutions, countries, or cultures.

### 2.3. Data Cleaning and Exploratory Data Analysis

Data cleaning was done to enhance the quality of data and make it suitable for machine learning analysis. The invalid values of CGPA recorded as 0 and Financial Stress recorded as “?” were considered missing values and were imputed with the help of median imputation since CGPA and Financial Stress were considered numerical and ordinal variables, respectively. The median imputation method was chosen to maintain the overall data distribution while minimizing the effect of extreme values [26]. The minor inconsistencies within the categorical labels were also made consistent before analysis, such as “Others” to “Other” in the Sleep Duration and Dietary Habits Variable.

Exploratory data analysis (EDA) was done through univariate analysis, bivariate analysis, and correlation analysis to study the distribution of features, class imbalance, the relation of predictors with depression, and the presence of multicollinearity. Correlation analysis was particularly done to check the presence of a strong interrelationship between numerical and ordinal predictors before developing the model. Most of the features had either weak or moderate correlations, which suggested the absence of multicollinearity and also that the retained predictors provided complementary information. Such analyses provided preliminary insight into the dataset and were used for feature selection, feature validation, and model development.

### 2.4. Feature Selection and Feature Validation

Feature selection was done so as to obtain variables that were relevant, had enough representation, and could be used for depression prediction, while features that were not relevant, very specific to the dataset, or had limited representation were not considered in the modeling process. These included ID, City, Profession, Work Pressure, and Job Satisfaction. ID was not considered since it did not offer any predictive value, while City was excluded to enhance the generalization of the model beyond the original geographical context. Profession, Work Pressure, and Job Satisfaction were removed because most records belonged to full-time students, resulting in very limited data for these variables.

The final feature set comprised Demographics (Gender, Age), Academic-related (Academic Pressure, CGPA, Study Satisfaction, Degree), Lifestyle (Sleep Duration, Dietary Habits, Work/Study Hours), and Psychosocial (Financial Stress, Family History of Mental Illness, Suicidal Thoughts) features. The selection of these features was informed by the data quality, their representation in the dataset, the relationship with risk factors of depression prediction, and alignment with previous studies on students’ psychological health [9,10,11,27].

The feature validation was done using sparsity analysis, permutation feature importance evaluation, and model comparison tests. Profession, Work Pressure, and Job Satisfaction were found to be the sparsest features. Out of the total 27,901 records, only 31 records contained information across 14 unique professions. Similarly, the Work Pressure and Job Satisfaction features had only 3 and 8 records, respectively. Using the permutation feature importance analysis, it was observed that these features had the least contribution among all the evaluated models. Further validation was also performed by comparing the performance of models before and after the use of the selected features. There were only minor changes in accuracy and ROC-AUC values across the models, with very limited predictive value. Since Suicidal Thoughts turned out to be the best predictor in the preliminary analysis, further validation was done by comparing the model performance with and without this feature. The feature was retained since it contributed to improving the predictive performance of all the evaluated models. These procedures were utilized to support feature selection rather than for establishing causal relationships.

### 2.5. Model Development

#### 2.5.1. Data Transformation and Feature Engineering

After performing data cleaning and finalizing the selected variables, the dataset was prepared to be employed in the machine learning algorithm. The categorical variable including Gender, Dietary Habits, Degree, Sleep Duration, Family History of Mental Illness, and Suicidal Thoughts were formatted suitable for machine learning analysis by using one-hot encoding. Academic Pressure, Study Satisfaction, and Financial Stress were kept as numerical variables because higher values indicate higher levels of the measured factor. Furthermore, Age, CGPA, Work/Study Hours, Academic Pressure, Study Satisfaction, and Financial Stress were standardized using StandardScaler to make them easier for logistic regression to process. The same data preparation steps, including imputation, encoding, and scaling, were applied consistently. These transformations were fitted only on the training data before being applied to the test data to minimize data leakage. Different one-hot encoding settings were used for the models. The logistic regression algorithm is highly prone to multicollinearity; thus, one category from each one-hot encoded variable was dropped using drop = first minimizing redundant information. On the other hand, both random forest and XGBoost algorithms used drop = none to retain all the encoded categories. Tree-based algorithms do not have such sensitivity toward multicollinearity and can effectively use all one-hot encoded categories without requiring a reference category to be removed.

#### 2.5.2. Machine Learning Models

Three supervised machine learning models, namely logistic regression (LR), random forest (RF), and Extreme Gradient Boosting (XGBoost), were built and evaluated to determine the most suitable for predicting the risk of depression among students. These algorithms were chosen as they represent diverse modelling techniques which are commonly used in machine learning research for education and health-related research, allowing predictive performance, fairness, and explainability to be assessed across both linear and tree-based methods [11,13,14,21].

Logistic regression (LR) was included as the baseline model because of its simplicity, computational efficiency, and interpretability. The model estimates the probability that a student belongs to the depressed class using the logistic sigmoid function:

(1) $$P\left(y=1|x\right)=\frac{1}{1+e^{-\left(\beta_{0}+\beta_{1}x_{1}+\cdots +\beta_{n}x_{n}\right)}}$$

where x1,x2,…,xn represent the input features such as Age, Academic Pressure, Sleep, and Financial Stress and the β represents the model coefficients that show how much each feature affects the prediction.

Random forest (RF) was selected as a tree-based ensemble learning algorithm that can handle complex nonlinear relationships while reducing overfitting through bootstrap aggregation. The final prediction is achieved by averaging the predictions generated by multiple decision trees:

(2) $$\hat{y}=\frac{1}{T}\sum_{t=1}^{T}f_{t}\left(x\right)$$

where ft(x) represents the prediction generated by each individual decision tree and T is the total number of trees in the ensemble. Random forest combines these three outputs to produce a more robust prediction for complex student data.

XGBoost was selected as a gradient boosting algorithm that sequentially builds decision trees, where each new tree learns from the errors of the previous trees to improve predictive performance. The ensemble prediction is expressed as:

(3) $$\hat{y}=\sum_{t=1}^{T}f_{t}\left(x\right)$$

where ft(x) represents the prediction contributed by each individual boosted tree. Unlike random forest, XGBoost builds trees sequentially, allowing each new tree to correct the errors of the previous trees and improve the overall prediction performance.

The hyperparameters of all three machine learning algorithms were fine-tuned by using five-fold GridSearchCV inside the training data. The most effective parameter setting for each individual algorithm was chosen based on the highest cross-validated ROC-AUC before evaluation on the independent test set, ensuring a fair comparison across all models.

#### 2.5.3. Model Training and Validation

An 80:20 train-test split was used, while ensuring stratification of the sample so that the initial ratio of depressed and non-depressed students is maintained in both subsets. This ensured that training and evaluating of models are carried out on representative data. Machine learning pipelines were employed to combine preprocessing and modeling into a single framework. This approach ensured that consistent procedures of preprocessing were being applied for all models, improving reproducibility and reducing the risk of preprocessing errors or data leakage. Since the data set was slightly imbalanced, class weighting was used to avoid the models from being biased towards the majority class.

Five-fold GridSearchCV was adopted to ensure an efficient evaluation of various combinations of hyperparameters. After determining the best hyperparameter configuration by means of GridSearchCV, each model was trained again on the entire training data set prior to testing performance on the held-out test set that was fully distinct from the training and optimization process. Such practice guaranteed an unbiased evaluation of how the models performed and its generalizability to unseen data. The process also facilitated the fair comparison of the performances of the models before evaluating their fairness, explainability analysis, and selecting the final model for prototype deployment.

### 2.6. Model Evaluation and Fairness Evaluation

Once the model was trained, its performance was analyzed using a held-out test set for evaluating its generalizability to unseen data. To conduct a comprehensive analysis of the model’s performance, various metrics were used for testing, such as accuracy, precision, recall, F1-score, ROC-AUC, and confusion matrices to provide a comprehensive assessment of classification performance. ROC-AUC and F1-Score have been given priority in the comparison of models since ROC-AUC determines the capacity of the model in distinguishing between students who are depressed and those who are not. Together, these two metrics give a more complete evaluation of model performance than accuracy alone.

Post hoc fairness evaluation was performed after the development of the models in order to determine whether predictive performance differed across gender and financial stress status. Logistic regression and XGBoost models were selected for fairness evaluation due to their high predictive performance among the models and represented two distinct modelling approaches: a traditional linear model and a more complex ensemble-based machine learning model. Separate evaluation of performance based on gender and Financial Stress groups, categorized as low (1–2), moderate (3), and high (4–5) was performed. The metrics were calculated independently for each group in order to check whether predictive performance is consistent. The analysis was designed to detect possible performance disparities across groups and did not involve fairness mitigation or modification of the trained models.

### 2.7. Explainability Analysis

Explainability analysis was performed using SHAP and LIME to improve the transparency of the selected XGBoost model. Logistic regression was included as an interpretable baseline, while the detailed post hoc explainability analysis was focused on the selected XGBoost model since it shows the best predictive performance. SHAP was used as the primary explanation method at different levels. The global explanation showed the most important features across the whole dataset, the class-level explanation showed how features influenced predictions toward depressed or not depressed, and the local explanation showed why the model made a specific prediction for one student. In contrast, LIME was used to provide additional local explanations for selected individual predictions. While SHAP was used to understand feature contributions at both the global and local levels, LIME was used to provide another perspective on individual cases and compare the explanations produced by the two methods.

Random forest and XGBoost used one-hot encoding internally, but in the SHAP local explanation, only the active category that matched the student’s actual answer was shown, instead of showing all encoded categories. Showing the active category made the SHAPs easier to read and understand, while keeping the same SHAP values that were calculated by the trained model. LIMEs were then compared with the corresponding local SHAPs to compare how the two methods attributed feature contributions for the selected cases. Since the study used observational secondary data, SHAP and LIME can explain which factors influenced predictions, but they cannot establish cause-and-effect relationships [24,28].

### 2.8. Prototype Development

In order to illustrate the feasibility of the proposed framework, an application using Streamlit was built. This application is based on the most successful model (XGBoost) and provides explanations of individual predictions using SHAP, which was chosen for the deployment because it offers both global and local explanations in one unified framework [11,14,29]. LIME was retained as a supplementary explainability technique and was applied primarily for comparison and validation when interpreting model outcomes. With the help of this prototype, the user will be able to input details about various attributes of the student’s demographics, academics, lifestyle, and psychosocial characteristics. After that, with the help of the XGBoost algorithm, depression risk will be predicted, and SHAP will provide the explanations. The prototype was developed as a proof-of-concept decision-support tool. It is intended to demonstrate how model predictions and explanations could be presented to users and is not intended for clinical diagnosis, independent decision-making, or direct intervention.

### 2.9. Reproducibility, Availability, and Ethical Considerations

Models were analyzed with Python (version 3.13.5) via Scikit-learn (version 1.6.1) and XGBoost (version 3.0.5), SHAP (version 0.47.2) and LIME (version 0.2.0.1) were used for explainability analysis, and Streamlit (version 1.45.1) was used to create the prototype application. Data utilized in this research can be accessed publicly through OpenML. The dataset used in this study is publicly accessible through OpenML. In order to maintain transparency and reproducibility, the data preprocessing, model training, hyperparameter tuning, evaluation, and prototype development procedures are documented in the thesis. Additional reproducibility information is provided in Supplementary Materials (Table S1).

As this study involved secondary analysis of a publicly available, anonymized dataset, no participants were recruited or directly involved, no interventions were conducted and no new data were collected. Therefore, there was no need for formal human participant ethics approval for the machine learning modelling stage. The research followed the Ngā Tikanga Paihere principles that ensured ethical and responsible usage of the data. These principles involved maintaining privacy by utilizing anonymized data, promoting transparency using explainable machine learning approaches through SHAP and LIME, and ensuring that the research centered on the wellbeing of students through risk detection rather than diagnoses.

## 3. Results

### 3.1. Predictive Performance and Model Selection

The predictive performance of logistic regression, random forest, and XGBoost on the test dataset is presented in Table 2. All three models achieved strong classification performance, with accuracy values ranging from 0.840 to 0.846 and ROC-AUC values above 0.90. XGBoost obtained the highest accuracy (0.846), recall (0.883), F1-score (0.870), and ROC-AUC (0.920), while logistic regression achieved the highest precision (0.879). random forest produced comparable results across evaluation metrics.

**Figure 2:**
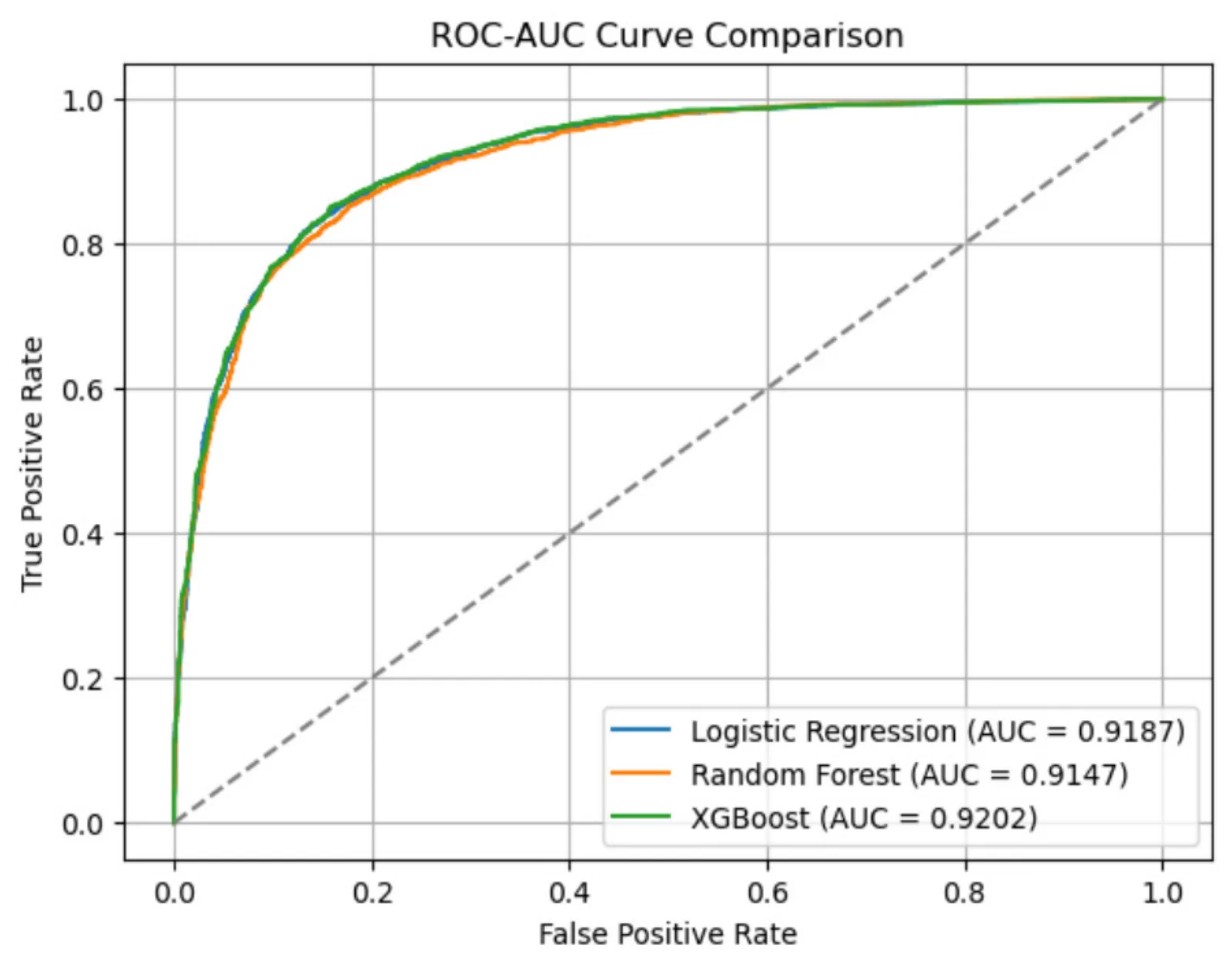
ROC-AUC curve comparison of logistic regression, random forest, and XGBoost. The dashed diagonal line represents the reference line for random classification (AUC = 0.5).

From the ROC curve in Figure 2, it is evident that all three models performed equally well in terms of discrimination performance. As the ROC curves were close together and were above the reference line, it can be seen that all three models exhibited excellent discrimination capability and were able to distinguish between depressed and non-depressed students. Although the best performing model was XGBoost, which performed with maximum accuracy, logistic regression performed similarly with this model in terms of accuracy and ROC-AUC. Thus, both models have been included to compare fairness outcomes across different modelling approaches.

Calibration was additionally assessed using the Brier score and calibration curves. XGBoost achieved the lowest Brier score (0.1096), followed by logistic regression (0.1128) and random forest (0.1149), indicating the best calibration among the three evaluated models. The calibration curves showed generally close agreement between predicted probabilities and observed outcome proportions. Detailed calibration results are provided in the Supplementary Materials (Figure S4 and Table S2).

**Figure 3:**
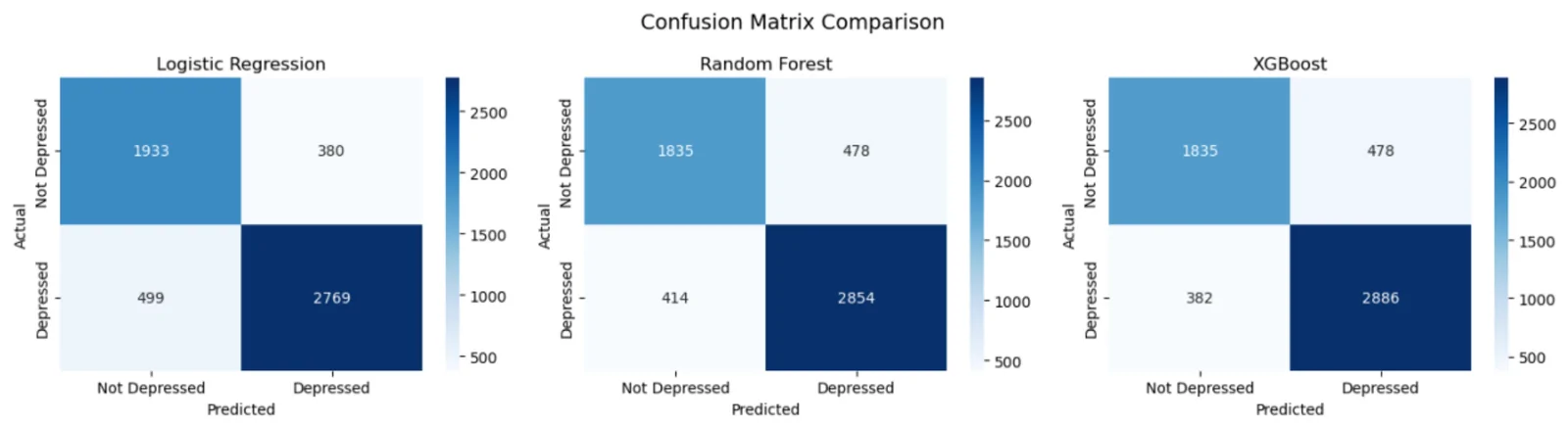
Confusion matrix comparison of logistic regression, random forest and XGBoost.

Figure 3 illustrates the confusion matrices of logistic regression, random forest, and XGBoost on the held-out test dataset. All three algorithms classified a significantly larger number of cases accurately than misclassified. The logistic regression model produced the least number of false positives (380), and this aligns with its highest measure of precision, whereas the XGBoost model had the least number of false negatives (382) together with the highest number of accurately detected depressed students (2886). These findings indicate a strong ability to detect students at risk of depression. Results obtained from random forest were close to those of both logistic regression and XGBoost and only performed slightly below XGBoost. These results are in line with the performance measures outlined in Table 2 and further confirm the selection of XGBoost as the best-performing model, while underlining the higher precision of logistic regression.

Feature importance and feature validation results proved that the final selected variables are appropriate to include in the final mode. Profession, Work Pressure, and Job Satisfaction have been omitted because of the lack of data obtained from the dataset, where the “Profession” variable is available for only 31 participants, and “Work Pressure” and “Job Satisfaction” contain 3 and 8 non-zero records correspondingly. The impact of using and not using these three variables was evaluated by testing the models, which showed little difference between accuracy and ROC-AUC implying that removing these variables did not significantly reduce model performance. Permutation feature importance further showed that the removed variables had very little impact on the model’s predictions. This approach is similar to what previous researchers have done wherein permutation feature importance was used to find variables that had low contribution to prediction and to reduce unnecessary model complexity [30].

**Figure 4:**
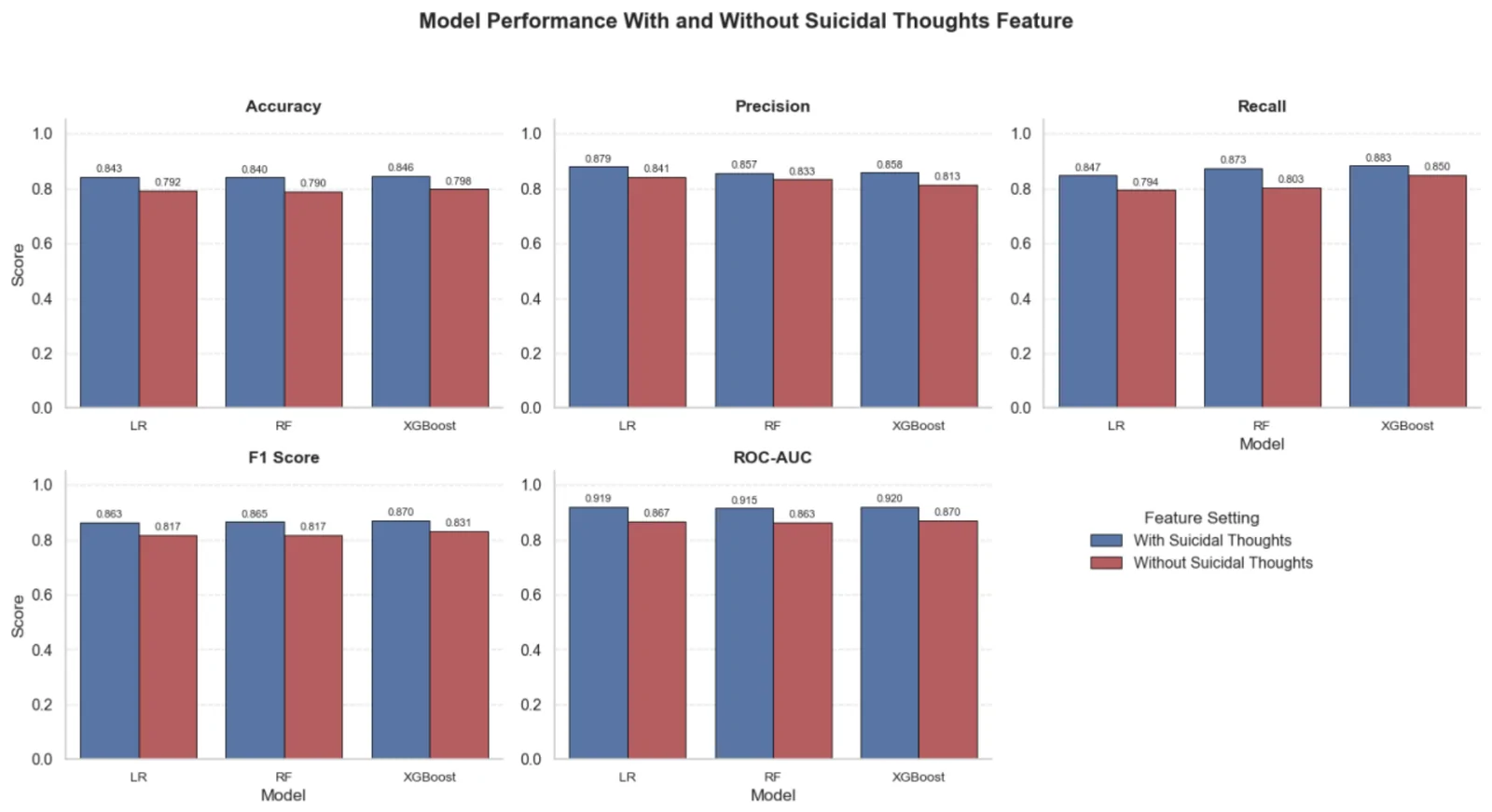
Performance comparison of logistic regression, random forest, and XGBoost with and without the Suicidal Thoughts feature.

While some variables contributed very little to prediction, Suicidal Thoughts consistently ranked as the most important feature across all models. Performance decreased across all models after its removal, demonstrating that Suicidal Thoughts contributed substantially to predictive accuracy. Overall, the validation results confirmed that removing Profession, Work Pressure, and Job Satisfaction did not harm model performance, while retaining Suicidal Thoughts improved predictive performance. To further evaluate the relevance of Suicidal Thoughts, the output was compared after the models were trained with and without this feature. Figure 4 shows the comparison of the values of accuracy, precision, recall, F1-score, and ROC-AUC across all algorithms used.

Across all models evaluated, the addition of *Suicidal Thoughts* has resulted in improvement in model performance. The ROC-AUC for logistic regression was improved from 0.867 to 0.919 and the accuracy from 0.792 to 0.843. Random forest also saw improvements with the ROC-AUC going from 0.863 to 0.915 and Accuracy from 0.790 to 0.840. Likewise, the XGBoost model saw improvements with its ROC-AUC from 0.870 to 0.920, and its accuracy from 0.798 to 0.846. Precision, recall and F1-score also improved across all models after including Suicidal Thoughts. The results suggest that Suicidal Thoughts played an important role in predicting depression risk. Therefore, it remained in the final modelling framework.

Notably, regardless of whether Suicidal Thoughts was present, XGBoost turned out to be the best performing algorithm. This indicates that the superior performance of XGBoost was due to its general capability to learn from the data, and not only because it used the Suicidal Thoughts variable. Logistic regression, random forest, and XGBoost model all showed success in predicting the probability of depression among students based on non-clinical variables. However, XGBoost proved to be the most successful of the three models by showing high accuracy, recall, F1-score, and ROC-AUC, while also producing the fewest false negatives. As a result, XGBoost was selected as the primary model for explainability and also for the Streamlit prototype. Nevertheless, both logistic regression and XGBoost models were used in the fairness evaluation.

### 3.2. Fairness Evaluation

A post hoc fairness evaluation was done using logistic regression and XGBoost in order to determine if the predictive performance of the two models differed across groups of gender and financial stress. The metrics used were accuracy, precision, recall, F1-score, specificity, false positive rate (FPR), and ROC-AUC. The analysis was intended to identify potential subgroup performance disparities and was not designed to establish comprehensive model fairness or perform fairness mitigation.

#### 3.2.1. Gender Fairness Result

**Figure 5:**
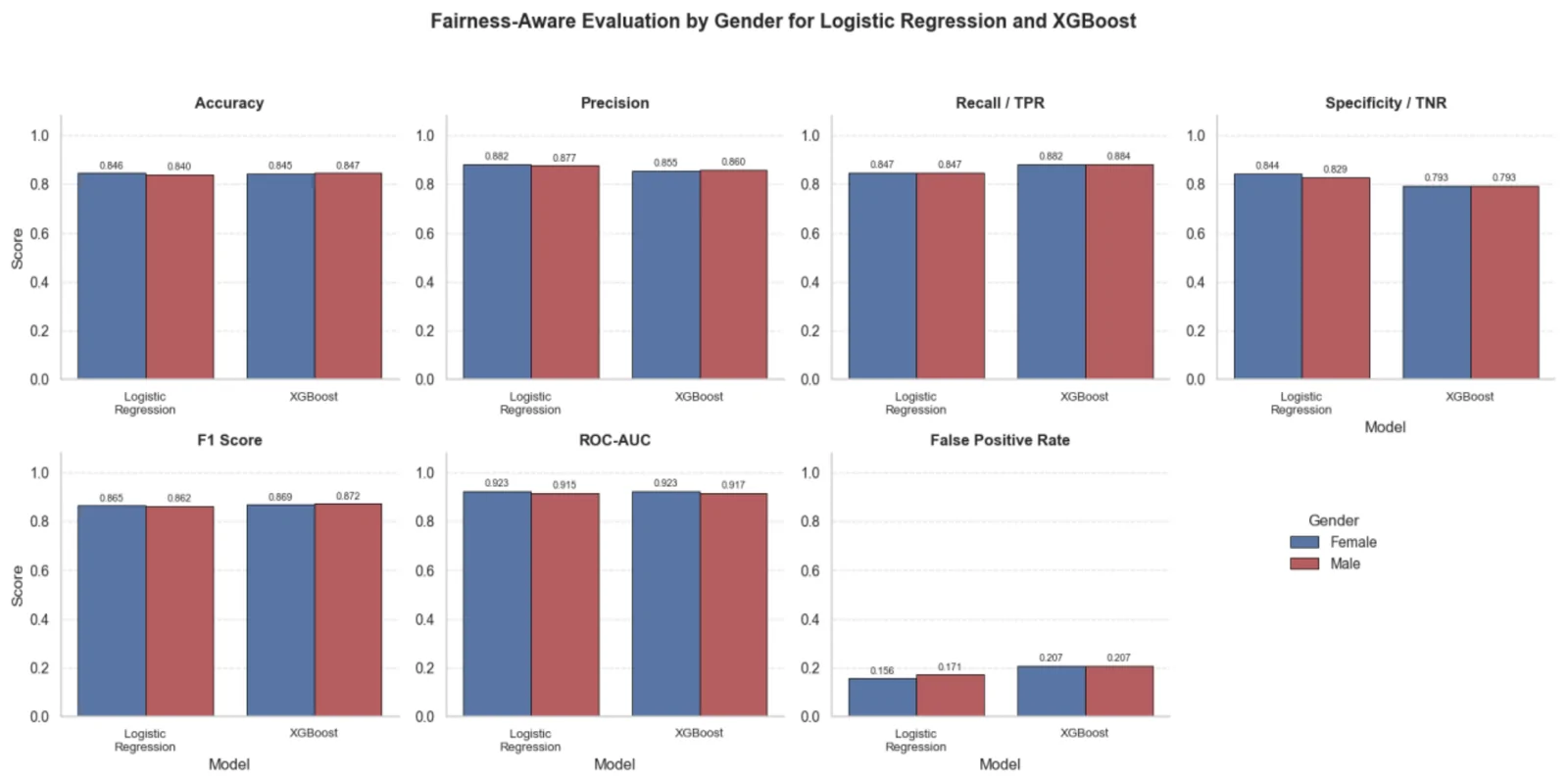
Fairness evaluation across gender groups for logistic regression and XGBoost.

Figure 5 provides the comparison on the performance of logistic regression and XGBoost models for male and female students to find out whether models of similar predictive performance occur across gender groups. There was very little difference between the values of accuracy, recall, F1-score, and ROC-AUC for both genders, meaning both models predicted risk equally across both groups. Logistic regression found an equal proportion of depression cases for both genders, whereas the XGBoost found an almost equal proportion with only a very slight difference. The false positive rate was noted to be nearly equal in both males and females, particularly for XGBoost where identical values were observed. Thus, no substantial differences were observed between male and female students, indicating relatively consistent performance across the evaluated metrics. However, this does not establish overall model fairness.

#### 3.2.2. Financial Stress Fairness Results

**Figure 6:**
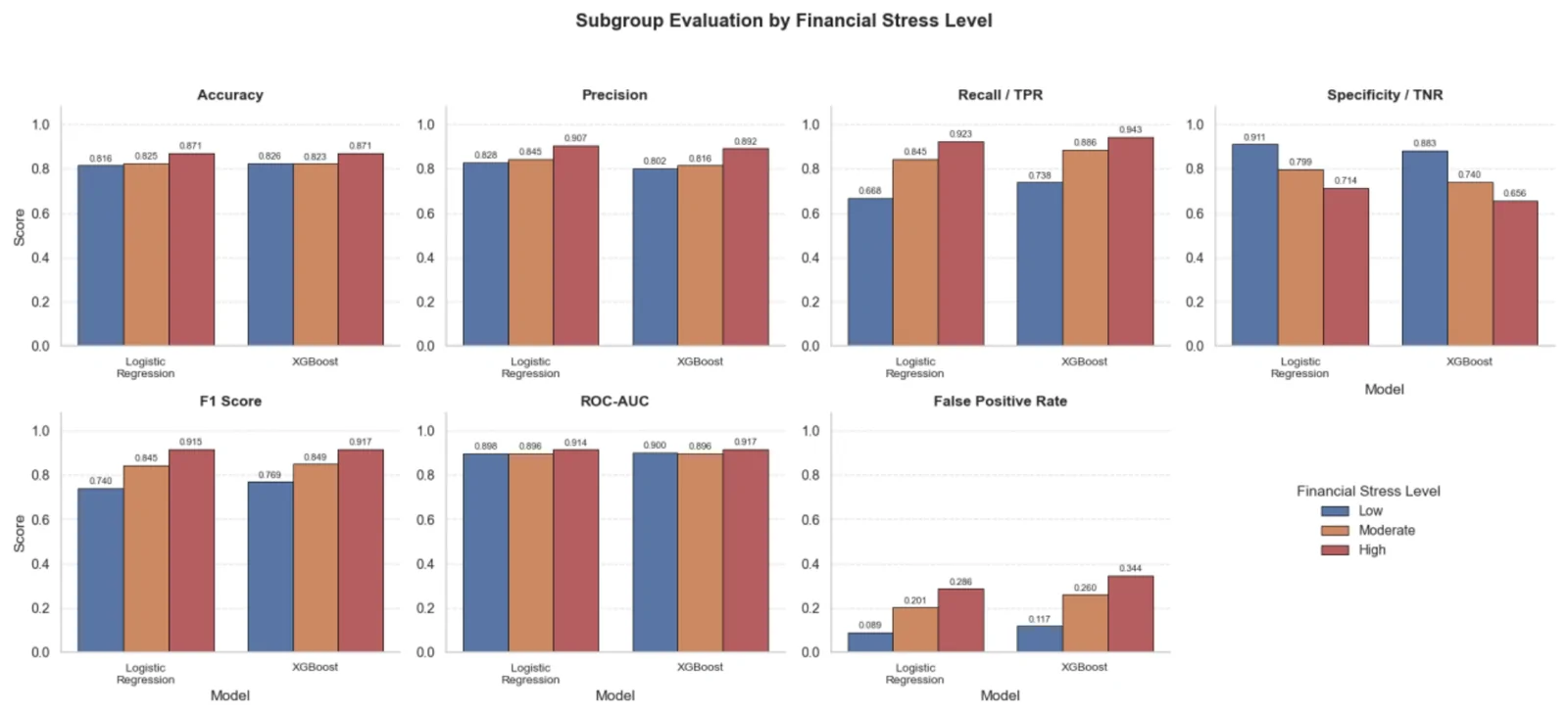
Fairness evaluation across low, moderate, and high financial stress groups for logistic regression and XGBoost.

Figure 6 presents the fairness evaluation results by comparing the performance of logistic regression and XGBoost across low, moderate, and high financial stress groups. Compared with the gender-based evaluation, larger differences in model performance were observed across financial stress groups. For both logistic regression and XGBoost, accuracy, precision, recall, F1-score, and ROC-AUC generally improved as financial stress levels increased. The largest difference was found in recall. For logistic regression, recall increased from 0.668 in the low financial stress group to 0.923 in the high financial stress group. For XGBoost, recall increased from 0.738 to 0.943. This means both models were better at correctly identifying depressed students in the high financial stress group than in the low financial stress group. Although recall improved as financial stress increased, specificity decreased and false positive rates increased, indicating that the models became more likely to incorrectly classify non-depressed students as depressed in the higher financial stress groups.

The results indicate that the models performed well in identifying students with depression in the group facing high financial stress; however, the models also made many errors or false positives when they predicted depression for those students who were not experiencing depression. These differences in subgroups may be partly attributable to differences in the depression prevalence among the financially stressed subgroups. Given that 75% of the students in the high financially stressed subgroup suffered from depression, as compared to 39% among those in the low financial stress subgroup, then depression-related patterns would be easily detected in the high-stress group. Overall, no clear performance disadvantage was observed between the models’ performances on the male and female subgroups; however, there were considerable differences on the financial stress groups. This illustrates the need for evaluating model performance on various subgroups rather than relying only on overall results.

### 3.3. Explainability Analysis Results

The explainability analysis was carried out to determine the factors affecting the prediction made by the XGBoost model. The use of SHAP and LIME helped to determine the features that increased or decreased the predicted depression risk. SHAP was employed to analyze the feature importance, across global, class level, and individual level, whereas LIME was used to develop local explanations for selected cases and see if it agrees with the explanations by SHAP.

#### 3.3.1. Global SHAP Analysis

**Figure 7:**
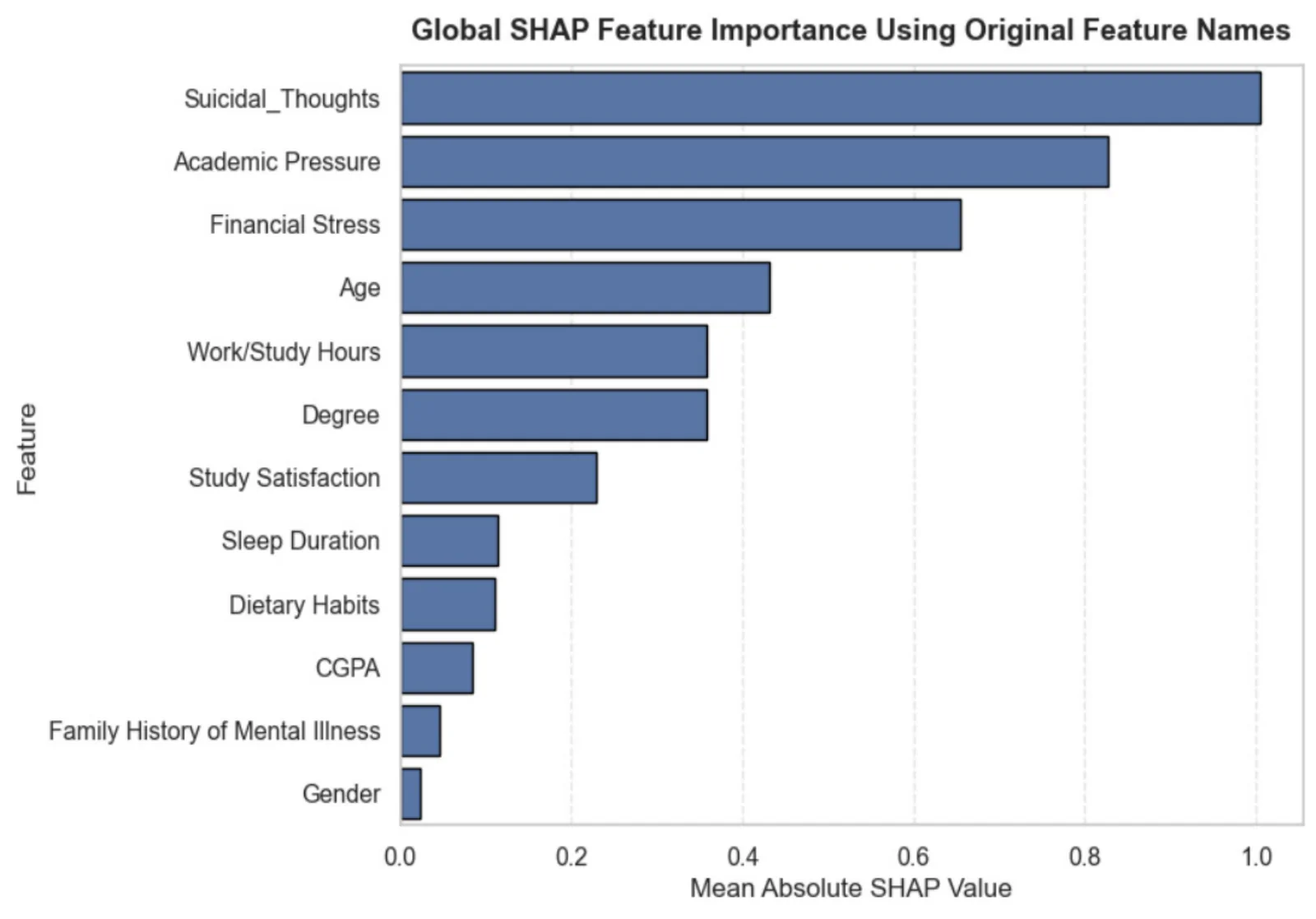
Global SHAP feature importance for the XGBoost model.

Figure 7 shows which features were most important to the XGBoost model when making depression risk predictions. The features were ordered from most important to least important according to their average SHAP importance.

As shown in Figure 7, among all the features used by the XGBoost model, Suicidal Thoughts had the greatest influence on the model’s predictions followed by Academic Pressure and Financial Stress. Age, Work/Study Hours, Degree, and Study Satisfaction also influenced the predictions moderately, whereas Sleep Duration, Dietary Habits, CGPA, Family History of Mental Illness, and Gender had a relatively smaller influence. Overall, the results suggest that psychosocial and academic-related factors were among the strongest contributors to the model’s predictions, although some demographic factors, particularly age, also influenced prediction outcomes.

#### 3.3.2. Class-Level SHAP Analysis

**Figure 8:**
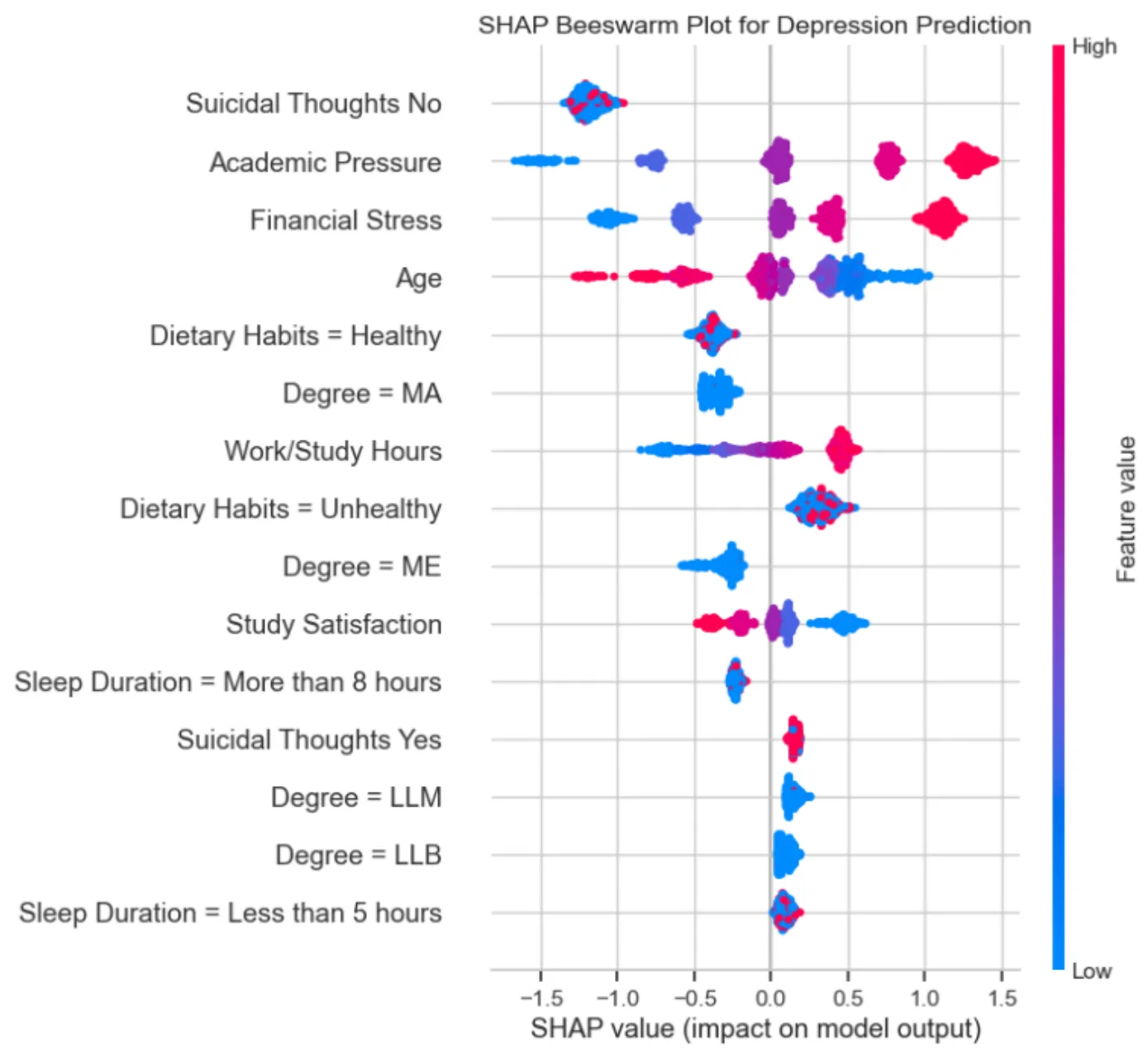
SHAP class beeswarm plot for the depression risk prediction using XGBoost.

Figure 8 presents the SHAP beeswarm plot, which provides additional insight into how feature values influenced depression risk predictions.

The class-level SHAP analysis was instrumental in demonstrating which factors led to predictions directed towards the high-risk class and which factors led to predictions directed towards the low-risk class. The identified features which had the most influence on the prediction of depression risk were Academic Pressure, Financial Stress, Suicidal Thoughts, Age, Work/School Hours, and Study Satisfaction. High levels of Academic Pressure, Financial Stress, and long hours at work/study contributed to the likelihood of predicting high risks of depression. On the other hand, older students and those with high Study Satisfaction were predicted to have low risks of developing depression. In addition, the analysis also revealed that Suicidal Thoughts had a strong influence on the outcome wherein the model was more likely to predict risk for depression among those who had Suicidal Thoughts. In conclusion, the results of the class-level SHAP demonstrated similar important factors as global analyses and revealed that the risk predictions for depression were significantly influenced by demographic, psychosocial, academic, and lifestyle-related factors.

#### 3.3.3. Local SHAP and LIME Analysis

**Figure 9:**
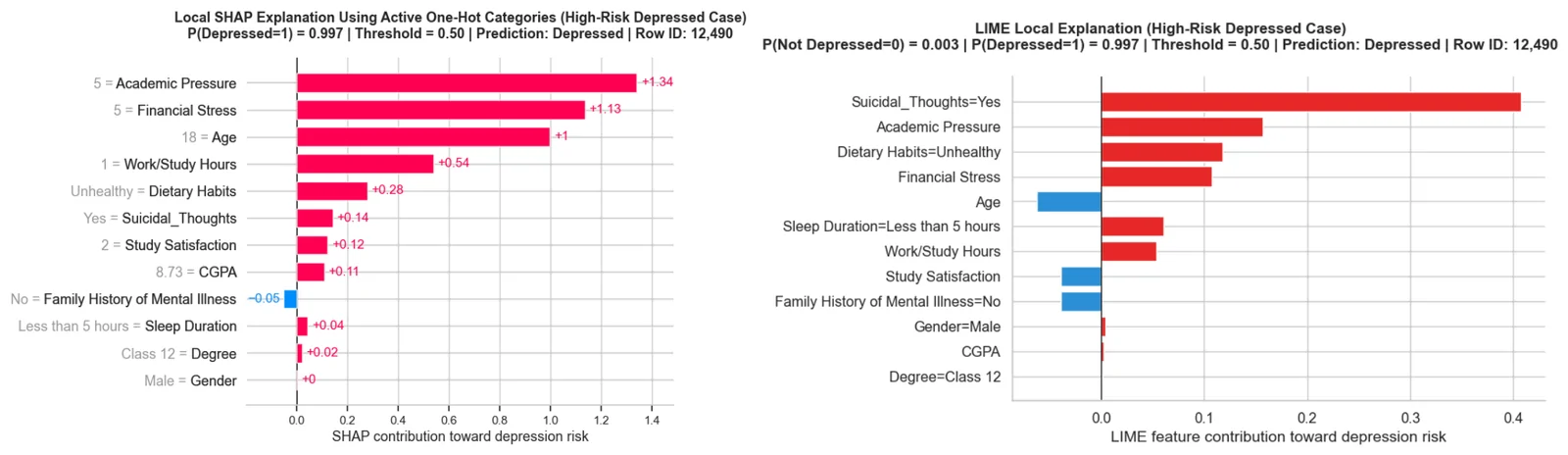
Shows local SHAPs and LIMEs illustrating key factors contributing to a high-risk prediction. Red indicates contributions toward higher depression risk, while blue indicates contributions toward lower depression risk.

**Figure 10:**
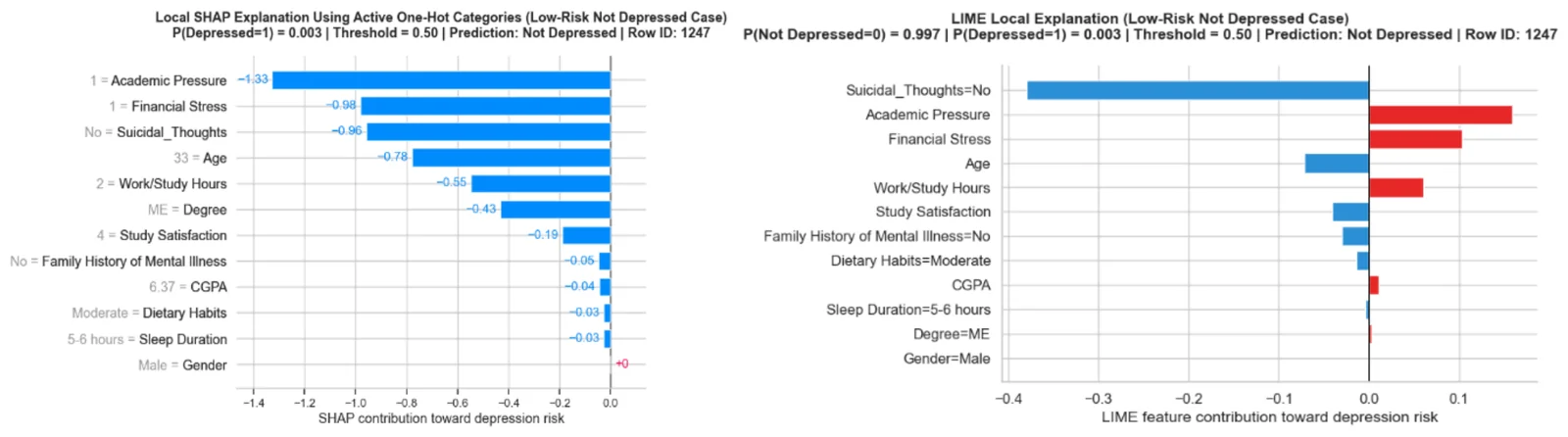
Shows local SHAPs and LIMEs illustrating key factors contributing to low-risk prediction. Red indicates contributions toward higher depression risk, while blue indicates contributions toward lower depression risk.

Figure 9 and Figure 10 presents the high risk/low risk local SHAP and LIME, which shows explanations illustrating key factors contributing to the predictions.

Local SHAPs and LIMEs were generated for two correctly classified individual cases to understand the factors influencing specific predictions and to assess the consistency between the two explainability methods. A student whose prediction was high risk and a student whose prediction was low risk have been selected, and the factors affecting their predictions using a SHAP and a LIME have been analyzed.

The variables which the SHAP and LIME identified as influencing the model to predict high-risk students include suicidal thoughts, academic pressure, financial stress, and lifestyle factors. On the other hand, the variables identified by both methods as contributing to the lowering of depression risk in the case of low-risk students included lower academic pressure, lower financial stress, and lack of suicidal thoughts. Although the SHAP and LIME did not present the exact same magnitude for each variable, both methods highlighted overlapping important factors.

The overlapping features identified by the two methods provided additional perspectives on the individual predictions, although their contribution magnitudes differed. Across both global and local analyses, Suicidal Thoughts, Academic Pressure, and Financial Stress were consistently identified as the most important factors influencing depression risk predictions.

An additional sensitivity analysis was performed by removing Suicidal Thoughts from the XGBoost model to examine whether other factors remained influential in the absence of this highly predictive variable. The removal of Suicidal Thoughts resulted in reduced predictive performance across all three models; however, XGBoost remained the best-performing algorithm, consistent with the main analysis shown in Figure 4. Global SHAP analysis showed that Academic Pressure became the most influential feature, followed by Financial Stress, Age, and Work/Study Hours. Local SHAPs and LIMEs for the same high-risk and low-risk correctly classified cases showed that Academic Pressure, Financial Stress, Age, and other academic and lifestyle factors continued to contribute to the predictions. These findings indicate that although Suicidal Thoughts noticeably influenced model predictions, the model continued to rely on multiple academic, financial, demographic, and lifestyle factors when this variable was excluded. Because Suicidal Thoughts is conceptually related to psychological distress, its strong predictive contribution should be interpreted cautiously and should not be taken as evidence of a causal relationship with the dataset-defined depression outcome. Detailed sensitivity-analysis results are provided in the Supplementary Materials (Figures S1–S3).

### 3.4. Prototype Demonstration

The Streamlit prototype demonstrated how the final XGBoost model and SHAPs could be integrated into an online web application, allowing users to view both the prediction and the factors influencing that prediction. The prototype lets the user input student demographics data, academic, lifestyle, and psychosocial data to get the predicted outcome of depression risk along with the probability score associated with it.

**Figure 11:**
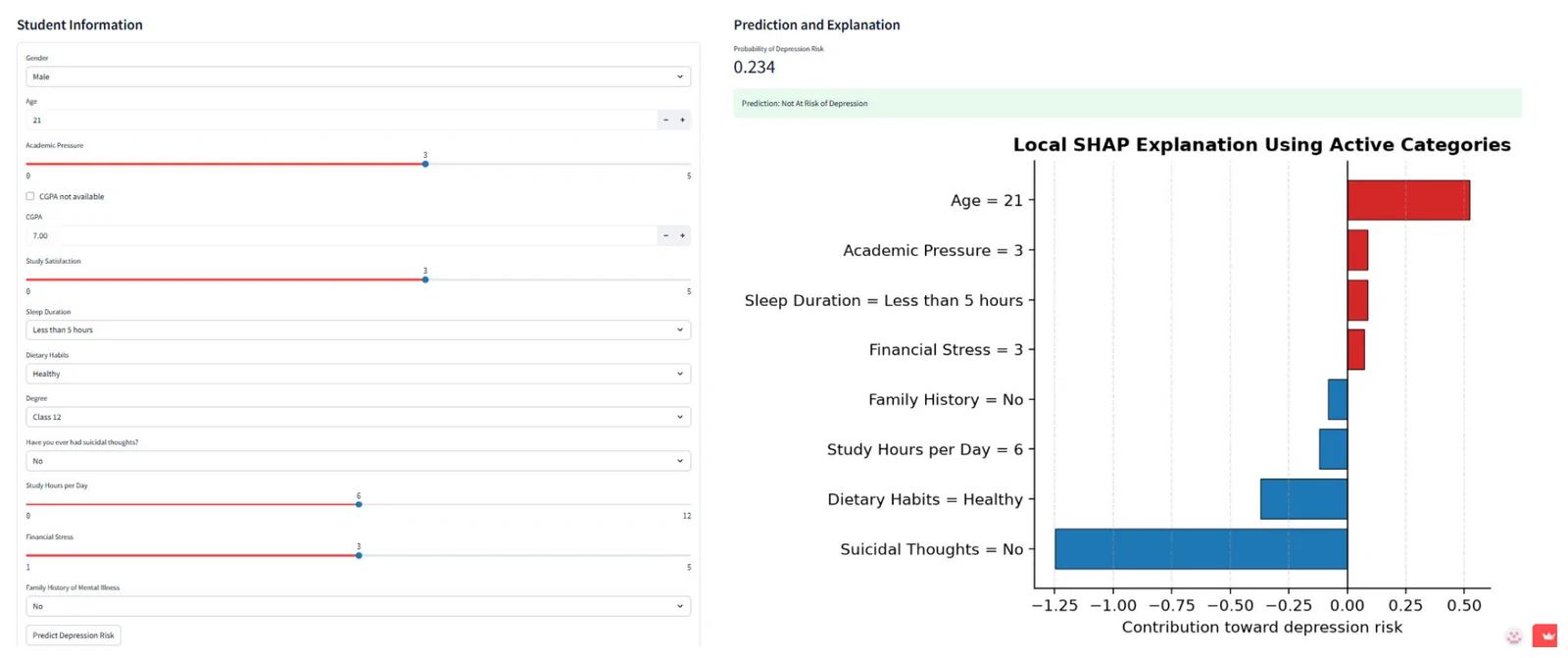
Streamlit-based student depression risk prediction system. Red indicates contributions toward higher depression risk, while blue indicates contributions toward lower depression risk.

Figure 11 presents the prototype interface using an example prediction. Along with the predicted risk category, the system shows the local SHAPs that indicate which input variables were increasing or decreasing the predicted risk. This allows the prediction output to be presented with an interpretable explanation rather than as an isolated classification result.

## 4. Discussion

### 4.1. Predictive Performance and Key Predictors

The study showed that the evaluated machine learning models achieved good predictive performance using non-clinical student information. Despite all of the models showing high levels of performance, XGBoost model demonstrated the best overall performance among others and, thus, was chosen as the final model. It should be noted that this result is generally aligned with previous research wherein good predictive performance of student depression predictions was found [9,11,13,14]. Table 3 shows a comparison between the performance obtained in the current study and selected earlier studies concerning prediction of student depression and mental health.

As shown in Table 3, the performance of the machine learning models in this paper was also similar to the performance shown by different studies on student depression and mental health prediction. The most relevant study for comparison is that of Li et al. [9], since the same dataset was utilized. Though the GLNet by Li et al. [9] performed slightly better with a 0.934 ROC-AUC, the XGBoost model in the present study showed a close performance with 0.920 ROC-AUC. Luo et al. [13], Zhang et al. [2], and Nath et al. [17] also obtained strong model performance in their studies. This indicates that machine learning may be a useful approach for identifying depression risk among students. However, any direct comparison between the studies should be made carefully as the studies adopted distinct datasets, features, pre-processing methods, target definitions, and model architectures. As opposed to Lamba et al. [19], as well as other previous studies which primarily concentrated on predictive performance, the present study also included fairness evaluation, explainability analysis, and a Streamlit prototype within the same framework.

Through feature validation, it was established that Suicidal Thoughts is a key predictor, since its presence in the analysis improves accuracy, recall, F1-score, and ROC-AUC for all considered models. It can be concluded that self-report measures of psychological wellbeing, like Suicidal Thoughts, may serve as significant predictors when combined with other demographic, academic, lifestyle, and psychosocial factors. Even though Suicidal Thoughts proved to be a highly significant predictor of the model, this does not imply that it is the cause of depression. The result should be interpreted as one factor associated with depression risk and considered together with other aspects of student wellbeing and mental health.

The explainability analyses provided additional insight by identifying the features that influenced the model’s predictions at both the global and individual levels. Both permutation feature importance (PFI) and SHAP consistently identified Suicidal Thoughts, Academic Pressure, and Financial Stress as the most influential predictors, while Age, Work/Study Hours, and Study Satisfaction also contributed to model predictions. The importance of Academic Pressure and Financial Stress in this study is similar to findings from previous studies, which have also shown that academic and financial challenges are strongly related to student mental health outcomes [10,11]. The findings are consistent with the Social Determinants of Mental Health perspective because several influential predictors, including academic pressure and financial stress, reflect social and educational conditions that may affect student mental wellbeing.

In summary, the research findings reveal that the non-clinical data of students is effective in predicting depression risk, especially those factors that are connected with the students’ academic and psychosocial wellbeing. The research findings further indicate that predictive models that utilize techniques such as SHAP and LIME supports both predictive performance and interpretability by identifying the factors most strongly associated with model outputs.

### 4.2. Fairness and Explainability Implications

While predictive performance is an important consideration, machine learning models used in educational and mental health applications should not be evaluated based on predictive performance alone. Their fairness across different groups and the transparency of their predictions should also be assessed. For this reason, the study also checked how the model performed for different groups of students and used explainability techniques to understand how the model made its predictions.

The fairness evaluation showed that model performance was relatively consistent across gender groups, meaning the models produced similar results for male and female students. However, unlike gender groups, the model’s performance was not the same across financial stress groups. The model generally performed better when predicting depression risk for students with higher levels of financial stress. Even though the differences between financial stress groups do not automatically mean that the model is unfair or biased, they show why it is important to evaluate how the model performs for different groups and not rely only on overall performance metrics. Previous studies have shown that high overall model performance can sometimes mask differences in performance between subgroups, highlighting the importance of fairness evaluation [20,21,22,31].

This difference in the performance of the model based on financial stress could be attributed to the difference in the prevalence rate of depression since the prevalence of depression was higher for students with higher financial stress levels. For instance, the prevalence of depression was 39% for low-stress levels and about 75% for high-stress levels. The model could have worked better in the group of higher financial stress since the signal for the depression was much clearer in this group, not necessarily due to any form of bias. It is essential to highlight the fact that the objective of conducting this fairness evaluation was to determine whether there was any disparity in performance in terms of the different groups, rather than fairness mitigation or to modify the trained models.

The explainability analysis made the framework easier to understand by showing how the model made its predictions. The overlapping results produced by SHAP and LIME increased confidence in the identified important features and showed that the model could be explained both overall and for individual students. This is particularly relevant in mental health applications, where understanding the factors that contributed to the prediction can help users interpret and appropriately respond to the result and is equally important with predictive accuracy.

In summary, the results for fairness and explainability reveal the importance of going beyond predictive accuracy in evaluating machine learning models. By integrating fairness evaluation and explainability analysis, the framework supports a more transparent and responsible approach to student depression risk prediction.

### 4.3. Practical Implications, Limitations, and Future Directions

The results from the study suggest that machine learning models can be used to identify students who may be at risk of depression at an earlier stage by using non-clinical factors. By relying on demographic, academic, lifestyle, and psychosocial information, it may support timely identification of students who could be at risk of depression. However, it is only intended to raise awareness and should not replace assessment or diagnosis by mental health professionals.

The deployment of the Streamlit prototype on Streamlit Cloud demonstrated that machine learning predictions and SHAPs can be integrated into an accessible web application for end users. The prototype not only tells users the predicted depression risk but also explains why that prediction was made in a way that is easier for non-technical users to understand. However, the prototype is only a demonstration tool. It can provide predictions and explanations, but it should not replace mental health professionals or make decisions on their own. Because Suicidal Thoughts are included as a model input, future implementation should include appropriate safeguarding, referral, and human oversight. The prototype is not intended to independently assess or manage suicidal risk or replace professional assessment.

While the findings demonstrate the potential of the proposed framework, several limitations should be considered when interpreting the results. The research involved the use of an already existing dataset of students belonging to one set of students, and the framework was not applied to a different independent dataset. Although 5-fold cross-validation was used for model selection, repeated nested cross-validation, bootstrap validation, and confidence intervals for the principal performance metrics were not performed. Hence, it remains uncertain whether the same results would have been obtained for students from other schools, countries, cultures, or populations. In addition, the model could only use the information that was available in the dataset. Some variables were removed because there were too few valid records for reliable analysis, while other factors that may be important for depression risk were not included in the dataset at all. Moreover, SHAP and LIME were capable of giving insights into why the model provided a certain output, but neither can help in understanding what factors are causing depression. Lastly, the prototype proved the feasibility of the proposed framework, but its usability and effectiveness were left unverified, as it was not tested on actual users; therefore, the prototype should not be used as a standalone tool for making decisions about students or determining appropriate mental health interventions.

Future research will need to overcome the shortcomings of this study by validating the model through other datasets in various educational settings. Further studies should also consider repeated nested cross-validation, bootstrap validation, confidence intervals, and external validation using independent datasets to assess model stability and generalizability. Future research will also need to test out the prototype with the appropriate stakeholders, including counsellors, support personnel, and students to assess usability, interpretability, and responsible use in practice. In addition, future work could extend the subgroup evaluation to other demographic and socioeconomic groups and incorporate formal fairness measures, confidence intervals, and appropriate mitigation strategies where meaningful disparities are identified.

Conclusively, all research objectives of this study were fulfilled by the findings obtained. XGBoost was identified to be the most efficient model that can predict the risk of student depression, fairness evaluation helped provide insights in the subgroup performance within gender and financial stress groups, the SHAP and LIME helped increase the explainability of models’ predictions, and the Streamlit prototype showed how the proposed framework could be used in a practical decision-support environment.

## 5. Conclusions

The purpose of this study was to develop an explainable machine learning framework for predicting student depression risk using non-clinical factors. To achieve this logistic regression, random forest and XGBoost models were developed and evaluated using demographic, academic, lifestyle and psychosocial variables. Furthermore, fairness evaluation, XAI techniques and a Streamlit-based prototype was developed to demonstrate the feasibility of presenting predictions and explanations through a web-based interface.

All three models showed strong predictive ability, but XGBoost produced the strongest overall results based on the evaluation metrics performed in the study. Suicidal Thoughts was identified as one of the most influential factors for prediction, and the model also relied strongly on Academic Pressure and Financial Stress. Age, Work/Study Hours, and Study Satisfaction also affected the prediction, but not as strongly. The post hoc subgroup evaluation showed that the model performed relatively similarly for male and female students. However, the performance was more varied when it comes to financial stress groups, with the model generally identifying depression risk more accurately among students with higher financial stress. These findings describe subgroup performance within the evaluated metrics and do not establish overall model fairness. These results show that evaluating a machine learning model involves more than just measuring accuracy. It is also important to assess whether the model performs consistently across different groups and whether its predictions can be clearly explained.

The study demonstrated that SHAP and LIME can improve the interpretability of machine learning models by explaining the factors influencing predictions at the global, class-based, and individual levels. In addition, the prototype showed how users can not only receive a depression risk prediction but also understand the reasons behind that prediction through explanations provided by the system. Although the prototype was developed as a proof of concept, it demonstrated that explainable machine learning models can be integrated into practical applications for supporting student wellbeing and early risk identification.

Overall, the study demonstrated that non-clinical information such as Academic Pressure, Financial Stress, Sleep Duration, and other factors can be utilized to predict student depression risk accurately, while at the same time, allowing the predictions to be explained and evaluated for fairness. The study combined several important components—prediction, feature validation, fairness evaluation, explainability, and prototype development—all in a single framework, contributing to the advancement of research on the responsible use of artificial intelligence in education and mental health. Future studies should test the framework on different datasets, include other factors that may affect student mental health, and evaluate the prototype with actual users to determine whether it is feasible for application in real-world settings.

## Figures and Tables

**Table 1:** Summary of variables used in the study.

Category	Variables
Demographic	Gender, Age
Academic	Academic Pressure, CGPA, Study Satisfaction, Degree
Lifestyle	Sleep Duration, Dietary Habits, Work/Study Hours
Psychosocial	Financial Stress, Family History of Mental Illness, Suicidal Thoughts
Target	Depression
Variables Removed Before Modelling	ID, City, Profession, Work Pressure, Job Satisfaction

**Table 2:** Performance metrics of the three models.

Model	Accuracy	Precision	Recall	F1 Score	ROC-AUC
Logistic Regression	0.843	0.879	0.847	0.863	0.919
Random Forest	0.840	0.857	0.873	0.865	0.915
XGBoost	0.846	0.858	0.883	0.870	0.920

**Table 3:** Comparison of predictive performance between the present study and selected previous studies.

Study	Focus Area	Best Model	Accuracy	ROC-AUC
Present Study	Student Depression Prediction	XGBoost	84.60%	0.920
Li et al. [9]	Student Depression Prediction	GLNet	88.84%	0.934
Luo et al. [13]	Student Depression Prediction	Random Forest	87.50%	0.927
Zhang et al. [2]	Student Mental Health Prediction	eXGBM	85%	0.932
Nath et al. [17]	Student Mental Health Prediction	Fine-tuned random forest	95.60%	0.950
Lamba et al. [19]	Social media/behavioural Depression Prediction	Random Forest	84.20%	0.880

## Data Availability

The dataset used in this study is publicly available from OpenML. The dataset is *Student Depression Dataset* by Jurado, I. C. Available online: https://www.openml.org/search?type=data&status=active&id=46753&sort=runs (accessed on 6 August 2025).

## Supplementary Materials

The following supporting information can be downloaded at https://www.mdpi.com/article/10.3390/bioengineering13091083/s1, Figure S1: Global SHAP feature importance for XGBoost without Suicidal Thoughts; Figure S2: Local SHAP and LIME explanations for the high-risk correctly classified case without *Suicidal Thoughts*; Figure S3; Local SHAP and LIME explanations for the low-risk correctly classified case without *Suicidal Thoughts*; Figure S4: Three-model calibration curves; Table S1: Reproducibility Information; Table S2: Brier scores; Table S3: Gender Fairness Evaluation; Table S4: Financial Stress Subgroup Evaluation.
