# Supplementary material for: Explainable Machine Learning for Predicting Student Depression Risk
Source: Bioengineering (Basel). 2026 Sep 18;13(9):1083. doi: 10.3390/bioengineering13091083 (PMC13603325; doi:10.3390/bioengineering13091083)
Supplement: Supplementary file 1 [file bioengineering-13-01083-s001.zip › bioengineering-4520976-supplementary.pdf]

Supplementary Materials

a. Explainability Analysis - Without Suicidal Thoughts

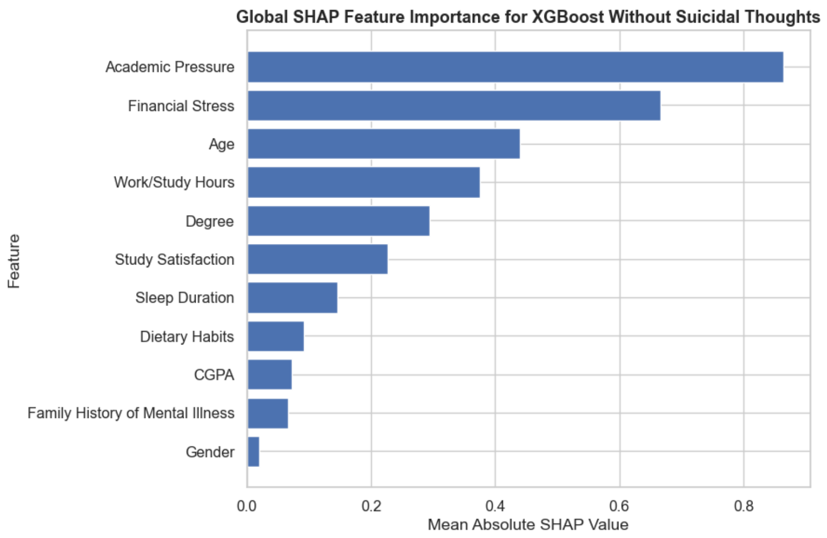

Figure S1. Global SHAP feature importance for XGBoost without Suicidal Thoughts.

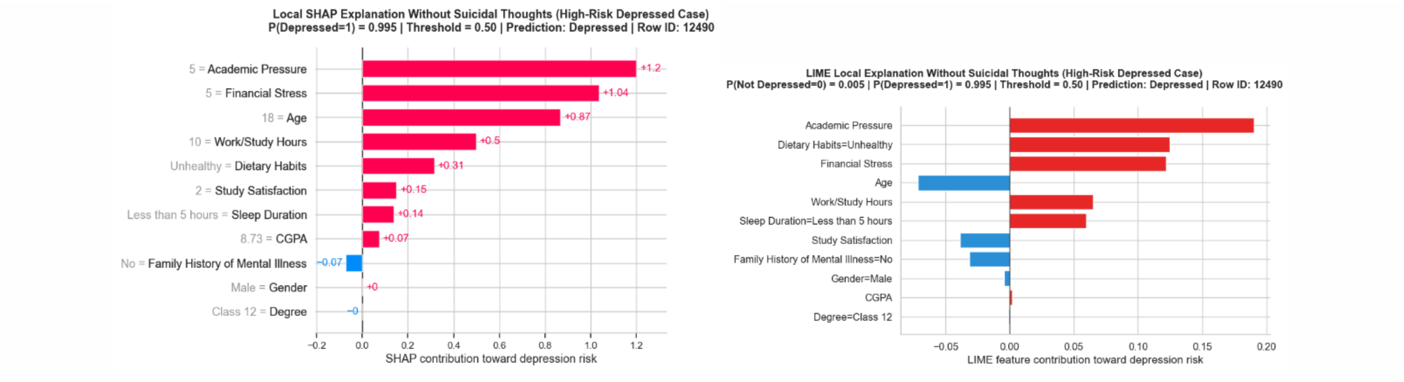

Figure S2. Local SHAP and LIME explanations for the high-risk correctly classified case without *Suicidal Thoughts.*

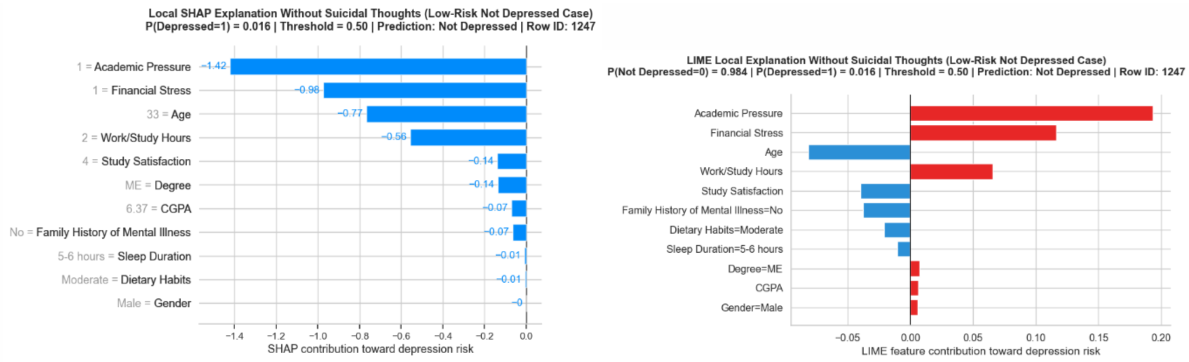

Figure S3. Local SHAP and LIME explanations for the low-risk correctly classified case without *Suicidal Thoughts.*

b. Reproducibility Information

Table S1. Reproducibility Information.

Item	Details
Dataset version	*student_depression_dataset*; Dataset ID: 46753; Version: 1
Missing-data proportions	12 missing values out of 334,812 cells (0.004%)
Random seed	42
Software/package versions	Python 3.13.5; pandas 2.2.3; NumPy 2.3.4; scikit-learn 1.6.1; XGBoost 3.0.5; SHAP 0.47.2; SciPy 1.16.3; Matplotlib 3.10.0
Hyperparameter search spaces - Logistic Regression	C= [0.01, 0.1, 1, 10, 100]; solver= [lbfgs, liblinear]
Hyperparameter search spaces - Random Forest	n_estimators=[100, 200]; max_depth=[None, 10, 20]; min_samples_split=[2, 5]
Hyperparameter search spaces - XGBoost	n_estimators=[300, 600]; max_depth=[3, 5]; learning_rate=[0.05, 0.1]; subsample=[0.8, 1.0]; colsample_bytree=[0.8, 1.0]
Selected hyperparameters - Logistic Regression	C=0.1; solver=lbfgs
Selected hyperparameters - Random Forest	max_depth=None; min_samples_split=5; n_estimators=200
Selected hyperparameters -XGBoost	colsample_bytree=0.8; learning_rate=0.05; max_depth=3; n_estimators=300; subsample=0.8
Class weights	Logistic Regression: class_weight='balanced'; Random Forest: class_weight='balanced'; XGBoost: no class weighting applied
Decision threshold	0.50
Subgroup sample sizes - Gender	Female: 2,471; Male: 3,110 test-set records
Subgroup sample sizes - Financial Stress	Low (1–2): 2,030; Moderate (3): 1,026; High (4–5): 2,524 test-set records
Analysis code	Available from the authors upon reasonable request
Deployed prototype	https://depressionriskprototype.streamlit.app/

c. Brier Scores and Model Calibration curves

Table S2. Brier scores.

Model	Brier Score
Logistic Regression	0.112827
Random Forest	0.114898
XGBoost	0.109625

https://doi.org/10.3390/xxxxx

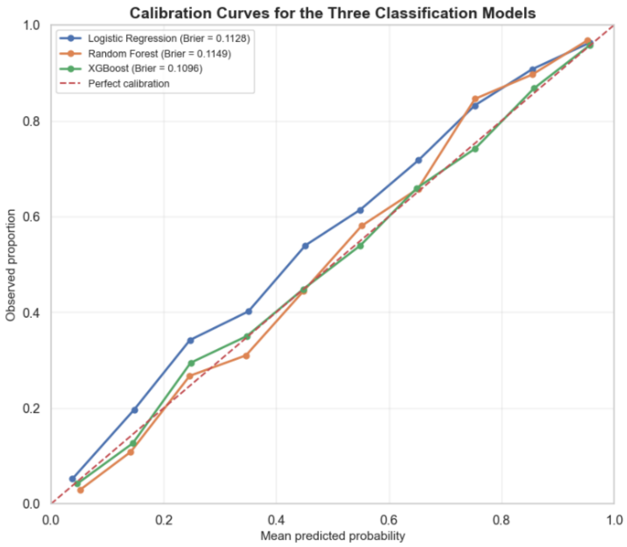

Figure S4. Three-model calibration curves.

d. Gender Fairness Evaluation and Financial Stress Subgroup Evaluation Table

Table S3. Gender Fairness Evaluation.

Model	Gender	Accuracy	Precision	Recall	Specificity	F1-score	ROC-AUC	FPR
LR	Female	0.846	0.882	0.847	0.844	0.865	0.923	0.156
LR	Male	0.840	0.877	0.847	0.829	0.862	0.923	0.171
XGBoost	Female	0.845	0.855	0.882	0.793	0.869	0.923	0.207
XGBoost	Male	0.847	0.860	0.884	0.793	0.872	0.917	0.207

Table S4. Financial Stress Subgroup Evaluation.

Model	Financial stress	Accuracy	Precision	Recall	Specificity	F1-score	ROC-AUC	FPR
LR	Low	0.816	0.823	0.668	0.911	0.740	0.896	0.089
LR	Moderate	0.825	0.845	0.845	0.779	0.845	0.896	0.201
LR	High	0.871	0.907	0.923	0.714	0.915	0.914	0.286
XGBoost	Low	0.826	0.802	0.738	0.883	0.769	0.906	0.117
XGBoost	Moderate	0.823	0.816	0.886	0.740	0.849	0.906	0.260
XGBoost	High	0.871	0.892	0.943	0.656	0.917	0.917	0.344
